# Integrated Genomic and Bioinformatics Approaches to Identify Molecular Links between Endocrine Disruptors and Adverse Outcomes

**DOI:** 10.3390/ijerph19010574

**Published:** 2022-01-05

**Authors:** Jacopo Umberto Verga, Matthew Huff, Diarmuid Owens, Bethany J. Wolf, Gary Hardiman

**Affiliations:** 1 Dipartimento di Scienze della Vita e dell’Ambiente, Università Politecnica delle Marche, 60131 Ancona, Italy; Jacopoumberto.verga@gmail.com; 2 School of Biological Sciences, Institute for Global Food Security, Queens University Belfast, Belfast BT9 5DL, UK; dowens13@qub.ac.uk; 3 Department of Medicine, Medical University of South Carolina, Charleston, SC 29425, USA; mlhuff92@gmail.com; 4 Department of Public Health Sciences, Medical University of South Carolina, Charleston, SC 29425, USA; wolfb@musc.edu

**Keywords:** Endocrine Disrupting Chemicals (EDC), DNA microarrays, RNA sequencing, Adverse Outcome Pathway (AOP), Molecular Initiating Event (MIE), Key Events (KE), risk assessment

## Abstract

Exposure to Endocrine Disrupting Chemicals (EDC) has been linked with several adverse outcomes. In this review, we examine EDCs that are pervasive in the environment and are of concern in the context of human, animal, and environmental health. We explore the consequences of EDC exposure on aquatic life, terrestrial animals, and humans. We focus on the exploitation of genomics technologies and in particular whole transcriptome sequencing. Genome-wide analyses using RNAseq provides snap shots of cellular, tissue and whole organism transcriptomes under normal physiological and EDC perturbed conditions. A global view of gene expression provides highly valuable information as it uncovers gene families or more specifically, pathways that are affected by EDC exposures, but also reveals those that are unaffected. Hypotheses about genes with unknown functions can also be formed by comparison of their expression levels with genes of known function. Risk assessment strategies leveraging genomic technologies and the development of toxicology databases are explored. Finally, we review how the Adverse Outcome Pathway (AOP) has exploited this high throughput data to provide a framework for toxicology studies.

## 1. Introduction

In the twentieth century a large quantity of contaminants, both organic and inorganic, has been released into the environment [1]. Many of these industrial chemicals have sufficient structural similarity to steroid hormones to be able to bind to steroid receptors or enzymes that regulate steroid hormone concentrations and, thus, perturb normal endocrine physiology in aquatic species, animals and humans [2,3,4,5,6,7]. Those chemicals capable of interfering with the endocrine system, mimicking the action of endogenous messengers (hormones) with their specific receptors are defined as endocrine disrupting chemicals (ECDs) [8]. This exposure of humans, fish and other animals to EDCs is a global concern and the subject of regulatory activity by various environmental agencies [4,9].

EDCs interfere with many hormone-regulated physiological pathways and have complex effects on human and fish physiology due to the diversity of the hormone receptors and enzymes that they bind [10]. The detailed mechanisms by which ED compounds act is not completely understood, but many interact with nuclear receptors, such as the retinoid X receptor (RXR) [11,12,13,14], peroxisome proliferator-activated receptors (PPARs) [14,15,16,17,18] and estrogen receptors (ERs) [19,20]. The binding of EDCs to these and other receptors, as well as steroidogenic enzymes, can perturb normal physiological processes. Health effects attributed to EDCs include reproductive dysfunction (e.g., distorted male/female sex ratios in fish, elevated or reduced hormone levels, diminished fertility, and male and female reproductive tract abnormalities) [21,22]; premature puberty [23]; neurological [24,25] and behavioral [26,27,28,29] complications; immune dysregulation [30,31]; cancer [32,33,34,35,36] and metabolic diseases [30,37,38,39,40,41,42,43,44,45,46]. EDCs include personal care products, plasticizers, detergents, insecticides, and pharmaceuticals. Many of these chemicals enter rivers and the ocean, where they directly interact with aquatic life and disrupt endocrine responses. Humans are exposed to these EDCs from drinking water and dietary consumption of fish [47]. In this review, we explore the major classes of EDCs, their health and environmental impacts and the emergence and application of high-throughput (HT) genomics technologies and bioinformatics approaches in studying their adverse effects. We explore how this has resulted in risk assessment strategies leveraging these HT approaches and the development of toxicology databases. We examine how the Adverse Outcome Pathway (AOP) framework leverages these data sets to determine the adverse health and environmental impacts.

## 2. Endocrine Disruptors

The threat from legacy contaminants lies in their ability to linger in the environment. In the past, the exposure of legacy contaminants was not well regulated because their effects on marine ecosystems and their potential to cause adverse outcomes, were not well characterized. As these discoveries were made, the random and careless dumping of chemicals was banned and more tightly regulated, but the concentrations of chemicals present in the environment have not necessarily decreased. For example, in the United Kingdom, the North Sea has seen a marked reduction in contaminant loads, but the concentrations of polychlorinated biphenyls (PCBs) found in local harbor porpoise blubber have not decreased in response [48]. Legacy contaminants are another term for what is known as persistent, bioaccumulative, and toxic (PBT) chemicals; these chemicals do not degrade easily and include toxins, such as cadmium, mercury, and PCBs [48].

A review of all endocrine disruptors is beyond the scope of this article, and we direct the reader to recent reviews on the future of toxicology testing for EDCs, in silico predictions of EDC properties and EDCs in teleosts [49,50,51]. In this section, we examine the major classes of EDCs.

### 2.1. Pesticides and Biocides

Particularly infamous among legacy contaminants are dichlorodiphenyltrichloroethane (DDT) and dioxins. DDT was widely used as an insecticide, due to its potency, from the 1940s to the 1970s. In 1962, conservationist Rachel Carson published her book Silent Spring, observing that DDT was bioaccumulating within the food chain and expressing her concern over the pesticide’s long-term effects on wildlife and humans [52]. By 1972, the use of DDT was banned in the United States; despite this, it can still be found in certain environments. The Palos Verde Shelf, located off the coast of Los Angeles, CA, remains profoundly contaminated by DDT, heavily linked to discharge of the chemical from the 1940s to the 1980s [53,54,55]. DDT is extremely hydrophobic, which leads to its accumulation in fat in the prostate, breast, and other tissues, where it can disrupt normal physiological responses. When applied to aquatic ecosystems DDT is quickly absorbed by organisms. Its breakdown products and metabolites, dichlorodiphenyldichloroethylene (DDE) and dichlorodiphenyldichloroethane (DDD), are also highly persistent and possess similar chemical and physical properties and can accumulate in the food web [56]. As with other hydrophobic compounds, DDT adsorbs to suspended particulate matter, and ultimately, is incorporated into bottom sediment [57,58]. Consequently, the Palos Verdes sediment serves as a sink for DDT and its metabolites, which can interact with fish and ultimately humans through the food chain in the Los Angeles basin [1].

Concerns about DDT, DDE and DDD effects on human health have led to monitoring programs for these legacy contaminants and remediation activities. However, thousands of additional chemical pesticides are being widely exploited today in domestic, agricultural, and industrial use. Many of these persist in the environment and are unfortunately toxic. As the production of these compounds will continue at least in the short term, environmental scientists are concerned about their short and long-term impacts on coastal and marine environments and their effects on human health.

Tributyltin (TBT) is one of the most widely dispersed EDCs in the marine environment where it was used as a biocide in anti-fouling paint. Humans, fish, and other animals are exposed to TBT and other organotins. The toxicity of TBT is such that the United Nations’ International Maritime Organization ratified legislation that bans the use of TBT in anti-fouling systems of ships. Tributyltin interacts with RXR and PPAR-γ [3] promoting the formation of fat cells and is an obesogen [3], a term describing any environmental chemical with the potential to alter metabolic pathways and make an individual more prone to obesity. TBT and other obesogens are of concern as causes of type 2 diabetes.

The dioxin 2,3,7,8-tetrachlorodibenzaodiolxin (TCDD) is widely dispersed in many ecosystems. TCDD is a persistent organic pollutant and was a contaminant in Agent Orange, an herbicide and defoliant chemical widely used during the Vietnam War. It interacts with the aryl hydrocarbon receptor (AhR) and is a carcinogen [59]. The AhR cross-talks with the ER, providing a link between dioxins and xenoestrogens (XEs) [60,61]. For example, TCDD induced hepatocellular carcinogenesis in females is estrogen-dependent [59].

### 2.2. Plastics and Microplastics

Microplastics have emerged as part of a whole new generation of chemical contaminants of emerging concern (CECs). Microplastics (<5 mm in size) are persistent, bioaccumulative, toxic chemicals (PBTs) that endure in the environment, are capable of long-range transport, biomagnify in food chains, and have been shown to significantly impact aquatic and human health. microplastics are now ubiquitous in shelf seas and coastal-marine regions [62]. Microplastics enter the marine environment directly through rivers, wastewater treatment plants (WWTPs) and carelessly stored sludge, beaches and coastlines, and atmospheric deposition, but also form through the continuous breakdown of larger plastics [63]. Depending on the sources microplastics can be divided into two main categories: primary microplastics that directly derive from industrial products (including cosmetic and plastic particles from industrial products), and secondary microplastics that derive from the continuous breakdown of larger plastics [63].

Microplastics follow a similar path to persistent organic pollutants, sorbing onto plankton and marine aggregates while propagating through food chains [64]. They are diffused and mixed, advected, sink to the sediment, and work their way into marine ecosystems and the food web [65,66,67]. They bioaccumulate and biomagnify in marine ecosystems, to the extent they are now considered to be of serious concern for aquatic and human health [62]. Microplastics uptake by marine organisms has profound consequences, directly leading to blockage of digestive tracts, false satiation, depletion of energy reserves, inflammatory responses, and developmental defects [68].

Five plastic contaminants found in micro and macroplastics that have received much attention are Bis(2-ethylhexyl) phthalate (DEHP), bisphenol A (BPA), 4-nonylphenol (NP) and di (n-butyl) phthalate (DBP) [4,6,7,69] (Table 1). DEHP, BPA, DBP and NP are found in plastics and can bind to the ER. Humans are exposed to these chemicals through leaching from plastic containers. Exposure of pregnant women to these chemicals may lead to endocrine-related abnormalities in the child later in life [6]. A concern is the potential of these chemicals to cause breast cancer [6,70,71].

Phthalate esters are used extensively in plastics and consumer goods including toys, which can be a source of phthalate exposure to children. DEHP is an example of a phthalate, the diester of phthalic acid and 2-ethylhexanol. It is a popular plasticizer, used to increase the flexibility of plastics, due to its low cost, its solubility in oil but not water, and its suitable chemical properties. In addition to DEHP, humans are exposed to DBP in high concentrations [21,72,73,74]. DBP down-regulates testosterone synthesis. DBP affects the synthesis of nearly 400 genes in Leydig cells, Sertoli cells and gonocytes [21,22]. As a result, the male reproductive tract in rats is adversely affected by DBP. DBP does not act by binding to the androgen receptor (AR). Transient exposure in embryos and in infants to these plastic EDCs impacts reproduction and development, in addition to promoting the development of endocrine-related cancers.

### 2.3. Perfluoroalkyl Substances (PFASs)

PFAS are used in many everyday items, ranging from food packaging, cookware, and toiletries, to clothing, carpets, cosmetics, and firefighting foams (Table 1). PFAS are forever chemicals’ owing to their long-term persistence in the environment, taking hundreds of years in some cases to degrade, allowing concentrations to accumulate over time. Subsequent resuspension and repartitioning during periods of strong currents and sorption to organic matter means that PFAS enter ecosystems and move up through the food chain, bioaccumulate and biomagnify at the various trophic levels, thereby posing a threat to ecosystem health and services [75,76,77].

The two most found contaminants are the perfluoroalkyl acids (PFAA), perfluorooctanoic acid (PFOA) and perfluorooctane sulfonate (PFOS), commonly referred to as legacy PFAS [78]. Collectively these persistent organic pollutants (POPs), many of which populate degrading coastal landfills provide a significant threat to the coastal ecosystem. Data from animal studies of PFOA indicate that exposure leads to the development of tumors [79,80]. Neonatal death has been linked with PFOA exposure [78,79]. Additionally, toxic effects on the immune, hepatic and endocrine systems have been reported [79].

### 2.4. Polychlorinated Biphenyls (PCBs)

Polychlorinated biphenyls (PCBs) are a class of organic compounds, widely employed in heat transfer fluids, coolants, electrical equipment and also as plasticizers given their resistance to chemicals and high temperatures [81]. The high chemical-physical resistance and lipophilicity have allowed its accumulation in the environment and access to the trophic chain, the main route of exposure for humans [82]. A wide range of human health effects have been observed in PCBs exposure: higher serum levels are associated with blood and immune cells reduction [83]. Metabolic disorders include type 2 diabetes, insulin resistance and obesity [84]. Neurobehavioral disorders include neuropathies, cognitive, motor, and sensory impairments [84]. Other severe effects include endocrine disruption and direct activity of the pollutants against sex hormone receptors [84,85].

### 2.5. Synthetic Estrogens

Ethinylestradiol (EE2), is a contraceptive that is found in municipal wastewater effluent discharges in the US and Europe [5,9]. EE2 is active at pM concentrations and can alter sex ratios in fish. A classic example of the deferred consequences of exposure to an EDC is seen with in utero exposure to diethylstilbestrol (DES) [72]. Females exposed transiently to DES during fetal development develop rare cancer as adults [72]. DES fetal exposure causes other toxic effects in women, in addition to adversely affecting male reproduction [72,86]. DES perturbs global gene expression across different tissues in males and females [72,87,88,89].

**Table 1:** EDC classes and detected levels in human bodily fluids.

Chemical	Class	Presence in Humans
BPA	Plasticizer	2.68–4.74 ng/mL urine, 1.56–1.71 ng/mL serum, 0.74 ng/mL breast milk, 0.53–12.7 ng/g placenta [90] 3.78 ng/g adipose tissue, 1.48 ng/g liver, 0.91 ng/g brain [91].
DEHP	Plasticizer	34.05 ± 26.00 ng/mL breast milk [92] 0.3–1.0 ppm adipose tissue [93].
DBP	Plasticizer	26.61 ± 18.03 ng/mL breast milk [92].
PFOA	PFASs	44.4 ng/mL serum [94].
PFOS	PFASs	3.9 ng/mL serum [94], 41.9 ng/g liver [95], 1.24 ng/g placenta [96].
DDT	Pesticide	162.95 ± 156.44 ng/g lipid (breast milk) [92], 0.969–3.710 mg/kg adipose tissue [97].
PCBs	PCBs	175.05 ± 85.22 ng/g lipid (breast milk) [92], 1.51–3.65 ng/g adipose tissue [97], 18.9–816 ng/kg adipose tissue [98].

Endocrine Disruptors, Environmental and Human Health Impacts. Bisphenol A (BPA), Bis(2-ethylhexyl) phthalate (DEHP), di (n-butyl) phthalate (DBP), perfluorooctanoic acid (PFOA), perfluorooctane sulfonate (PFOS), dichlorodiphenyltrichloroethane (DDT), and polychlorinated biphenyls (PCBs).

## 3. Environmental and Human Health Implications of EDC Exposure

As noted above, the structural conformation of EDCs allows them to mimic natural E2. E2’s biological role is to regulate sexual differentiation and reproduction, though it also plays roles in tissue differentiation, inflammation control, and neurological functions [99]. These compounds that mimic E2 are called xenoestrogens (XEs) and impact normal estrogen signaling pathways, resulting in adverse health outcomes in aquatic and terrestrial species and humans [47].

### 3.1. Impacts on Aquatic Species

#### 3.1.1. Fertility

The primary function of estradiol (E2) is related to sexual characteristics and reproduction, so the main concern with exposure to XEs is how it impacts these processes. As an analog of E2, EE2 provides a clear example of how a XE can alter reproductive processes. An in vivo study in the fry of rainbow trout revealed that exposure up to 10 μg/L EE2 enriched pathways related to the cell cycle and DNA replication, and demonstrated the ability of EE2 to disrupt gonad differentiation [100]. While sex differentiation in fish is more labile than in mammals, the ability of EE2 to alter transcriptional patterns during female gonad differentiation in male fish demonstrates the estrogenic effects of EE2 [100].

NP has weak estrogenic activity and can inhibit the binding of E2 to the ER, interfering with the homeostasis of natural hormones. Studies of 100 μg/L exposure of NP have indicated reduced testis size, decreased sperm number, and infertility, among other adverse outcomes, as the result of long-term exposure to NP in teleost [101].

An experiment with goldfish (*Carassius auratus*) treated with DEHP concentration up to 100 μg/L indicated that starting from 10 μg/L exposure to the phthalate reduced sperm quality and kinetics (i.e., motility and velocity), the result of disrupted pituitary and testicular hormonal functions [102].

#### 3.1.2. Cancer

RNA-Seq of the Korean rose bitterling (*Rhodeus uyekii*) indicated that exposure to 100 ng/L of EE2 for seven days induced expression of apoptotic genes, including p53 and TNF-α, and cancer genes, including *Smad2* and *CAV2* in hepatic and skin tissues [103].

#### 3.1.3. Metabolic Disease

In male zebrafish (*Danio rerio*), 4 ng/L EE2 exposure for 90 days post-fertilization was related to the build-up of adipocytes in the testis, suggesting signs of early obesity [40]. An in vitro study on the livers of brown trout indicated that EE2, as with other estrogenic compounds, interfered with the activity of PPAR-γ, with a significant (3-fold) decrease in response to 50 µM EE2 [15]. In adult male ten spotted live-bearer fish (*Cnesterodon decemmaculatus)*, exposure to a concentration of EE2 up to 200 ng/L was associated with increased steatosis, necrosis, and disruption of acinar organization (effects detected starting from 100 ng/L EE2) [41].

NP can accumulate within the liver and has been connected to adverse changes in its metabolic processes. Exposure from 2 to 220 μg/L to NP is connected to increased expression of hepatic PPAR-α and PPAR-β [104]. These increased levels of PPARs suppress the expression of cytochrome P450 isoforms (CYP1A1 and CYP3A4) needed for the liver to complete detoxification pathways. In Italian newts (*Lissotriton italicus*), exposure to 50 and 100 μg/L nonylphenol ethoxylates (NPE) is associated with the development of large lipid droplets within their livers [42].

Exposure to 5.8 nM DEHP has been associated with the activation of pathways related to the development of the metabolic disorder Non-Alcoholic Fatty Liver Disease (NAFLD) in zebrafish (*Danio rerio)* [44].

#### 3.1.4. Neurological Effects

The effects of EE2 on the neural development of fish have become well defined in recent years. One study utilized RNA-Seq to determine the effects of exposure to 8 ng/L and 38 ng/L EE2 in the neural transcriptome of both male and female guppies [24]. Their findings determined that EE2 altered transcript abundances in a sex-specific manner and that exposure to EE2 had a feminizing effect on the male transcriptome.

### 3.2. Health Impacts in Animals

#### 3.2.1. Fertility

Exposure to both environmentally relevant and toxicological levels of EE2 (0.1 and 1 μg/kg bw∙day respectively) in female mice between embryonic day 10 and postnatal day 40 promoted advanced vaginal opening and shorter estrus cycles [105].

In female rats, dietary exposure to BPA (oral gavage 0.001, or 0.1 mg/kg for 90 days) is connected to a decreased concentration of E2 found in the ovaries, and alterations in the expression of steroidogenic acute regulatory protein (StAR) and P450 aromatase, two proteins with fundamental importance to ovarian steroidogenesis [106]. The authors of this study concluded that prolonged exposure to BPA reduced E2 levels, leading to increased apoptosis of ovarian cells. Male rats treated with 100 mg/kg body weight of nonylphenol, BPA, or a mixture of both the compounds showed similar effects including delayed puberty, testicular damage, and reduced spermatogenesis. Moreover, BPA exposure damaged kidneys causing hydronephrosis [107].

Similar phenotypes caused by DEHP exposure were found in both fish and mammals; Sprague-Dawley rats treated with DEHP (oral gavage of 250, 500, 750 mg/kg over a 30-day period) displayed decreased testicular sperm count, and reductions in daily sperm production (DSP), and serum testosterone levels [108]. Further studies in mice have indicated that these changes are the result of epigenetic changes in mouse sperm cells [109]. DEHP induces methylation-associated silencing of seminal vesicle secretory proteins and antigen genes, which play fundamental roles in the physiology of sperm cells, and increases the expression of mir-615 miRNA [109]. Female rabbits treated with DEHP (1 mg/kg/day) showed increased functional luteolysis, which could undermine the luteal support in the ovarian cycle [18].

Experiments regarding DBP exposure in male rats observed significant adverse effects on fertility [74,110,111]. Prenatal DBP exposure (100 and 500 mg/kg body weight) led to decreased sperm count, motility, and viability. Other effects included altered testicular structure and decreased testosterone serum levels in exposed rats [110]. Pubertal DBP exposure in male rats (from 0.1 to 500 mg/kg day) significantly reduced testes and epididymal weight, with dose-dependent histological changes observed in the testes [74].

#### 3.2.2. Cancer

In Agouti mice, maternal exposure to BPA decreased CpG methylation upstream of the Agouti locus, shifting the distribution of the color of the animals within progeny [112]. It is important to emphasize that the Agouti study established the concept that epigenetic modifications in the mother may be passed down to the next generation [112].

Studies in pregnant rats treated with EE2, and subsequently treated with the carcinogen 9,12-dimethylbenz[a]anthracene (DMBA) (oral gavage 10 mg at 50 post-natal day) to induce mammary tumorigenesis, indicated that the elevated estrogen levels caused by EE2 elevate the risk of breast cancer cells developing resistance to tamoxifen (TAM) and the risk of local recurrence [113]. Similarly, long-term exposure to EE2 (0.01 mg/kg bw∙day) in mice, prenatally and after sexual maturity, has been associated with the development of endometriotic and precancerous lesions in the uterus and ovary [32].

In rat liver epithelial WB-F344 cells, exposure to NP (from 2.2 μg/L to 22 mg/L) was associated with enhanced cell motility, as well as an increase in lysophosphatidic acid (LPA) receptor 3 (*Lpar3*) expression [114]. In cancer, cell motility is considered a potential target for preventing tumor metastasis and invasion, and the ability of NP to upregulate motility supports its ability to promote metastasis.

#### 3.2.3. Metabolic Disease

Long-term exposure to BPA in mice (5, 50, 500 and 5000 μg/kg bw∙day) enhances lipid accumulation, increases adipose serum mass and serum cholesterol levels, and the development of hyperglycemia and hypercholesterolemia [38].

Prenatal exposure to NP in pregnant rats (intragastric administration of 200 mg/kg bw·day) resulted in lower litter weight and fewer newborn rats compared to control rats, with newborns exhibiting increased serum and hepatic NP levels [115]. Additionally, the production of TNF-α, PCNA-, and IL-β in hepatocytes was increased within the NP-exposed group of newborn rats, indicating a link between chronic NP exposure and inflammation of the liver [115].

Rats treated with a high-fat diet and exposed to DEHP for 8 weeks (from 0.05 to 500 mg/kg) exhibited liver inflammation suggesting that DEHP accelerates the progression of NAFLD [116].

DBP treatments in pregnant rats (injection of 40 mg/kg bw∙day) significantly increased the expression of phosphorylated STAT1 and suppressed FoxM1, suggesting a crucial role of DBP in the development of gestational diabetes [117].

#### 3.2.4. Neurological Effects

In vivo exposures in rats demonstrated that maternal exposure to BPA (oral gavage 0.05, 0.5, 5 or 50 mg/kg bw∙day) is associated with impairment of object recognition in male offspring [27]. Another neurological pathway altered by exposure to BPA is the stress response; exposure to the EDC (oral gavage 40 μg/kg bw∙day) increased DNA methylation of glucocorticoid receptor regulator *Fkbp5*, an important regulator of the stress response, and ablation of estrogen receptor β (*ERβ*) reversed these effects [20]. Furthermore, prenatal exposure to BPA in male rats (oral gavage 2 mg/kg bw∙day from gestation day 10 to lactation day 7) is linked with anxiety and depression-like behaviors [28]. A study in perinatally exposed male rats indicates that BPA can reprogram the hypothalamic-pituitary-adrenal (HPA) axis to become hyperactive, the most constant trait associated with anxiety and depression [28].

Treatment with EE2 in pregnant rats (oral gavage 0.01, 0.1 or 1 μg/kg bw∙day) resulted in the offspring experiencing mild growth retardation and a dose-dependent increase in gonadotrophin-releasing hormone (GnRH-1) neurons; it should be noted that division of GnRH-1 neurons was not impacted by EE2 treatment [118].

NP has been identified as a potential neurotoxin, having been shown to induce apoptosis in mouse neurons by suppressing the anti-apoptosis protein bcl-2 and up-regulating caspase-3, a key executor of apoptosis (100 and 200 mg/kg bw∙day) [119]. Recent studies have identified the xenobiotic receptors retinoid X receptor (RXR), pregnane X receptor (PXR), and constitutive androstane receptor (CAR) as mediators of NP-induced apoptosis in mouse hippocampus cells [11].

In general, DEHP is known to induce apoptosis in neurons. It has recently been shown that exposure to DEHP (7.8, 156.2 and 3124.5 ng) induces the expression of an oxidative stress enzyme, hemeoxygenase 1 (HO-1), in neuronal cells [120], and further research identified HO-1 as potentially responsible for this increase in apoptosis [121]. Prenatal exposure to the phthalate produced a marked decrease in cell proliferation and neurogenesis (for 1 and 100 mg/kg/day) in the neocortex of fetal mice, with an increase in cell death for 500 mg/kg [25]. Furthermore, these new-born exposed to DEHP showed both a decrease in the number of neurons and abnormal distribution of neurons. Intriguingly, DEHP exposure (0, 1, 10, or 20 mg/kg) impaired normal development of the male hippocampus, but not that of the female [122]. In males, expression levels of 52 miRNAs decreased after exposure in a dose-dependent manner, while 19 miRNAs increased in exposed females, indicating that while DEHP interferes with male hippocampal development, it promotes it in females.

#### 3.2.5. Asthma

Exposure to EDCs has been linked to the development of asthma and other allergic diseases. CD-1 mice orally exposed to 1 mg BPA with ovalbumin displayed enhanced eosinophil recruitment in the alveoli and airway submucosa, coupled with increased goblet cell proliferation in the bronchial epithelium [123]. They also displayed increased levels of Th2 cytokines-interleukin-13 (IL-13), and eosinophil-related cytokines including IL-5, and CCL2, indicating that exposure to BPA induces a Th2-mediated inflammatory response [123].

In a murine model of asthma, exposure to NP (5 and 500 μg/kg bw∙day) in both bone-marrow derived dendritic cells (BM-DCs) and splenic classic dendritic cells CD11c(+) cDCs secreted increased levels of IL-6 and TNF-α, at both baseline and following lipopolysaccharide (LPS) stimulation [124]. Furthermore, NP-exposed mice developed a more severe, ovalbumin (OVA)-induced allergic lung inflammation compared to wild-type mice, and these altered effects on DC function were not observed in mice with a low-affinity mutant AhR. What is most concerning about NP, and DEHP, is their presence in PVC tubing used for respiratory therapy; if EDCs are responsible for the development of asthma, their presence in these treatment options may only exacerbate a patients’ symptoms [125].

Finally, Zhou and colleagues looked at the effects of long-term DBP exposures (40 mg/kg bw/day) on asthmatic allergic mice [126]. Their results revealed a significant increase in inflammatory cell infiltration and typical asthma symptoms, including changes in lung histology [126]. Other effects included augmented oxidative stress and CGRP neuropeptide levels [126].

### 3.3. Health Effects in Humans

#### 3.3.1. Fertility

BPA exposure is connected with the loss of fertility in both males and females [127,128]. A study of 191 men with different rates of fertility revealed that BPA levels in seminal fluid were negatively associated with sperm count, concentration, and morphology, indicating that BPA negatively impacts sperm quality [128].

Exposure to NP in males is associated with negative impacts on spermatogenesis and sperm quality, leading to decreased fertility [129]. A large study involving 1590 men (877 infertile men and 713 controls) determined the levels of phenols in the urine and semen of adults, and identified NP as having a strong association with male infertility [130].

#### 3.3.2. Cancer

In the context of adverse outcomes, BPA exposure in human breast cancer MCF7 cells, and the mammary glands of six-week-old mice increased the expression of Enhancer of Zeste Homolog 2 (*EZH2*), a histone methyltransferase strongly associated with breast cancer. This increased expression resulted from increased histone H3 trimethylation at lysine 27, the primary histone modification catalyzed by EZH2 [112]. Research exploring the relationship between EE2 exposure and prostate cancer development is limited. Nevertheless increased levels of E2 are known to increase susceptibility to the formation of prostatic lesions with aging [33].

NP (220.35 μg/L), in addition to BPA (228.29 μg/L), has been shown to induce the epithelial-mesenchymal transition (EMT) through an ER dependent pathway in ovarian cancer cells, stimulating their migration and metastasis [19]. In the context of prostate cancer, an examination of the effects of NP on non-tumorigenic prostate cells revealed estrogen-like activities; at 10^−6^ M NP promoted a cytoplasm-nucleus translocation of ERα, as well as enhancing expression of key cell cycle regulator genes [131]. Furthermore, in both experiments, these phenotypes were reversed by the addition of the estrogen antagonist ICI 182,780, confirming the estrogenic effects of NP.

Treatment of the MCF7 breast cancer cell line with low concentrations of phthalates, including DEHP (10 nM), has been shown to increase their viability, coupled with increased expression of p-Akt and phosphatidylinositide 3-kinase (PI3K) [132]. This study also indicated that phthalates, when combined with E2, have an additive effect on improving MCF7 viability, increasing *Bcl-2* and *ERα* expression. In healthy human cells, DEHP induced DNA damage, leading to modified apoptosis and mitosis, increased cell proliferation, and elevated tumor mobility and invasiveness [34]. Furthermore, DEHP exposure was associated with inhibited gap junction communication and tight junctions, as well as the promotion of EMT. Due to the widespread usage of DEHP, it has become a concern for workers exposed to concentrations of phthalate via inhalation [133].

Low-dose DBP exposure revealed enhanced proliferation and expression of cell cycle genes (including cyclin-D, *CCND1*, Proliferating Cell Nuclear Antigen, *PCNA*, and cyclin-dependent kinase 4, *CDK4*) on prostate (PC3 and 22RV1) (10^−6^–10^−7^ M DBP) [35] and breast (BG1) cancer cell lines (10^−5^–10^−8^ M DBP) [36]. Epithelial cells (MCF10A) co-cultured with ER (+) and ER (−) breast tumor fibroblasts revealed a different response to DBP exposures (10^−8^–10^−7^ M) [134]. Only those cells co-cultured with ER (+) fibroblasts showed a significant increase in proliferation [134]. These results suggest the key role of ER expression in XE-induced carcinogenesis.

#### 3.3.3. Metabolic Disease

BPA’s effects on metabolic processes are well characterized and are known to lead to an increased chance to develop obesity and diabetes. BPA is classified as an obesogen [37]. In human adipose stromal/stem cells (ASCs), exposure to at least 1 µM of BPA significantly enhanced adipogenesis [135] and increased the expression of the ER (*ESR1*). Treatment with the ER antagonist ICI-182780 blocked the effects of BPA, indicating that BPA promotes adipogenesis through an ER-mediated pathway [136]. Urinary analysis of 1860 American children, ages 8–19, indicated that high levels of BPA are associated with higher lean body mass index z-scores in males (*p* < 0.05). High levels of BPA are associated with higher fat mass index z-scores in females (*p* < 0.05) [39].

Characterized by the accumulation of large lipid droplets within the liver, NAFLD has become the most frequent liver pathology in developed countries [137,138]. HepG2 cells treated with micromolar doses of DEHP (from 5 to 100 μM) promoted the expression of hepatic *PPAR-α* and sterol regulator element-binding protein 1c (*SREBP-1c*) [43]. Moreover, DEHP treatment increased oxidative stress and apoptosis indicating that it is hepatotoxic [43]. Further research has implicated exposure to DEHP (from 0.05 to 500 mg/kg) with deregulation of insulin signaling in rats and humans via PPAR-γ activation, diminishing the liver’s capacity to maintain glucose homeostasis and contributing to insulin resistance [116], ultimately leading to adverse outcomes, such as chronic liver disease, type 2 diabetes, and cardiovascular disease [139,140,141].

#### 3.3.4. Neurological Effects

A study of childhood exposure to BPA in the Cincinnati, Ohio, area revealed that children with higher concentrations of BPA in their urine displayed greater anxiety and poorer emotional control, with more notable changes observed in females [26].

In the context of human health, NP exposure is linked with the development of attention deficit hyperactive disorder (ADHD) [29].

#### 3.3.5. Asthma

Though human studies are limited, a recent study determined that the mothers of infants with allergic conditions had higher levels of urinary BPA in comparison to those who did not, and higher concentrations of BPA resulted in a higher risk of allergies in their children [142]. Exposing human, fetal lung fibroblasts to 100 µM of BPA appears to alter the expression of immune and developmental modulators, which may negatively impact fetal lung maturity and response to environmental stress [31].

Treatment of the human bronchial epithelial cell lines BEAS-2B and HBE135-E6E7 with NP inhibited their proliferation by promoting the Fas/Fas ligand apoptotic system [143,144]. Treatment of bronchial smooth muscle cells (BSMCs) with the culture media (CM) of BEAS-NP and HBE-NP induced the expression of inflammatory cytokines IL-6 and IL-8, subsequently inducing their proliferation and migration; these are major features involved in asthma remodeling [145].

As mentioned above, DEHP is used in the tubing of products designed for asthma treatment [125]. In Belgium, urinary analysis of 418 14–15-year-old adolescents for phthalate metabolites indicated an increased risk of an asthma diagnosis with the presence of DEHP metabolites in their urine [146]. Furthermore, increased levels of phthalate metabolites were correlated with increased levels of urinary 8-hydroxydeoxyguanosine (8-OHdG), a marker of oxidative stress, with a stronger association in girls, compared to boys. Further studies have potentially identified DEHP’s epigenetic modification capabilities as the cause; children with high urinary levels of 5OH-MEHP exhibited lower methylation of the TNF-α promoter region, and TNF-α methylation was inversely correlated with TNF-α protein levels [147]. To strengthen these connections, lower methylation of the 5’CGI region of TNF-alpha was associated with a higher risk of asthma in children. In addition, 56 Korean children with asthma were evaluated for lung function and the urine samples were tested for the presence of DBP metabolites [148]. Interestingly, the decrease in lung function has been associated with an increase in phthalate metabolite levels [148].

## 4. Genomics and Bioinformatics Approaches in Endocrine Disruptor Research

Over the past two decades, the field of ecotoxicology has adapted genomics technologies to better understand how exposure to compounds perturbs gene expression on a molecular level, facilitating transcriptome analysis. Historically, two tools have been used for this purpose: DNA microarrays and RNA sequencing (RNA-Seq) although microarrays are becoming an obsolete technology [149]. A challenge is to comprehend the effects of these chemicals on the health of humans as well as other animals. Much research has been carried out elucidating what concentrations of EDCs are toxic for regulatory purposes. Critical for obtaining this information is the identification of biomarkers and genomic signatures of the response to different chemicals in humans, fish, and other animals. This is a challenging task because the actions of each chemical are complex, involving the regulation of many genes and interactions between many physiological pathways. The methods for investigating the molecular effects of exposure to EDCs must sample a broad molecular response. A special concern regarding the effects of EDCs is that exposure at different stages in life: embryonic, postnatal, juvenile, and adult are likely to have different effects. Thus, although molecular analyses taken soon after exposure to a chemical are useful in diagnosis the response to a chemical, it also is necessary to perform these analyses over a generation and if possible, for more than one generation, to uncover delayed effects. A good example is the previously mentioned DES exposure which manifests in cancer development many years following the exposure and even affects later generations.

Genomics technologies have enabled the assessment of the effects of xenobiotics in the environment on human health [150]. Ecological exposure and risk assessment models alone, however, are not adequate to examine the effect of EDCs [150]. Producing exposure-to-outcome databases from diverse ecotoxicogenomic datasets and executing systems toxicology approaches, are required to comprehensively explain the risk of these toxicants and to assuage the ambiguities that currently exist with risk assessment. Recent work in sediment and dredged material assessment has advocated for the inclusion of biomarkers in acute bioassays [151]. Although chemical analyses have enabled the development of standards for regulating contaminant occurrence in the environment, there is a need for sensitive assays that can detect their bioavailability in fish, animals and humans. Chronic exposure to compounds with endocrine targets, particularly at low chemical levels, has been shown to disrupt vertebrate development [3,6,152,153]. Recent advances in molecular methods, have substantially improved the sensitivity for assessing the biological effects of CECs on animal physiology [2,154,155,156], but more pertinent, these tools provide chemical signatures of bioavailability and exposure and allow the development of risk assessment databases.

### 4.1. RNA Sequencing

From 2005, several massively parallel sequencing technologies termed Next-Generation Sequencing (NGS) emerged, resulting in increased throughput and accuracy, and reducing sequencing costs to less than a thousand dollars for a human genome. These high throughput parallel DNA sequencing platforms launched a new era of genomics and molecular biology. A key application is RNA sequencing (RNA-Seq) [157]. With RNA-Seq, the RNA is extracted from the biological samples and converted to cDNA [157] capturing the sequences and abundance of all mRNA transcripts at a given time point. RNA-Seq can be used to obtain per-base expression profiles [157], with several advantages when compared with microarrays including better sensitivity for those genes that are expressed at low or very high levels, splice variants, and non-coding transcripts (such as miRNA and lncRNA) [157,158].

Microarray analyses of the transcriptome have waned in the past five years as there are limitations to this method compared with RNAseq: the inability to detect novel transcripts that do not have specifically designed probes, the appearance of cross-hybridization artifacts in analyses of similar sequences, and decreased accuracy for transcripts present only at low levels [159,160,161,162,163,164,165]. Sequencing-based approaches to transcriptome profiling overcome these issues and are designed to directly determine transcript sequences [161]. In comparison to microarrays, RNA-Seq has minimal background technical noise. The sequence reads can be unequivocally mapped to unique regions in the genome and the technology has a greater dynamic range to quantify gene expression level. RNA-Seq can produce highly reproducible results, leveraging both biological and technological replicates, and requires less input RNA material [166,167,168].

Some of the drawbacks of RNAseq have been centered on data management, ease of use, and the total number of databases provided by both the scientific community and commercial vendors [169]. HTS experiments generate FASTQ files, massive raw data files containing the nucleotide sequence and quality score information. Considered “raw” data, FASTQ files are subject to secondary analysis, often including alignment to a reference genome or de-novo assembly, which generates secondary and intermediate files as massive as the initial, “raw” data. These derived files, in turn, may be stored, filtered, annotated, or analyzed in several ways that generate more data; in short, storage and organization of RNA-Seq data provide a challenge. In terms of the user experience, many current HTS tools require knowledge of intricate command-line instructions for their operation. This provides a considerable barrier for nontechnical audiences—who have little experience in computer science—from effectively utilizing RNA-Seq for their analyses. Finally, the number of databases and knowledge bases is notoriously expansive and increases in size every year; the most recent count, according to Nucleic Acids Research, is 1641 databases as of January 2021 [170]. Keeping up with these databases is a daunting challenge, and recent efforts have focused on improving these standards of knowledge sharing [171].

### 4.2. RNAseq for the Study of Endocrine Disruption

Leet et al. investigated the effects of early life exposures to the herbicide atrazine or EE2 on sexual differentiation and gene expression in gonadal tissue [172]. They used largemouth bass (*Micropterus salmoides*) from 7 to 80 days post-spawn to concentrations of 1, 10, or 100 µg atrazine/L or 1 or 10 ng EE2/L and monitored histological development and transcriptomic changes in gonad tissue. They noted an almost 100% female sex ratio in fish exposed to EE2 at 10 ng/L, likely as a result of sex reversal of males [172]. Not surprisingly many gonad genes were differentially expressed between the sexes.

Wang et al. examined the effects of 17α-methyltestosterone (MT), an artificial androgenic compound, used to induce masculinization of both secondary sex characteristics and gonads in aquatic studies. The Stone moroko (*Pseudorasbora parva*) was exposed to MT, and the growth and development of fish were delayed by exposure to MT at 200 ng/L. RNAseq analyses revealed 7758 and 11,543 DEGs in females and males. MT had more obvious disruption effects on males than females, and this was primarily reflected in the immune system [173].

Renaud et al., examined the effects of EE2 exposure on the Pacific sardine (*Sardinops sagax*) and chub mackerel (*Scomber japonicus*). RNA sequencing (RNAseq) was performed on liver RNA harvested from wild sardine and mackerel exposed for 5 h under laboratory conditions to a concentration of 12.5 pM EE2. This revealed that environmental levels of EE2 disrupted basic biological processes and pathways in both male sardine and mackerel, leading to molecular signatures of metabolic, hormonal and immune dysfunction, as well as carcinogenesis in exposed fish [174].

Bertucci and colleagues examined chronic exposure to wastewater treatment plant and stormwater effluents at the whole-transcriptome level in the Asian clam (*Corbicula fluminea*) and evaluated the physiological outcomes. They uncovered a set of 3181 transcripts with altered abundance in response to water quality. The largest differences in transcriptomic profiles were observed between clams from the reference clean site and those exposed to wastewater treatment plant effluents. Most of the differentially expressed transcripts were involved in signaling pathways involved in energy metabolism suggesting an energy/nutrient deficit and hypoxic conditions in response to the pollutants in the effluents [175].

Legrand and co-workers exposed copepods (*Eurytemora affinis*) to sublethal concentrations of the pesticide pyriproxyfen (PXF) and insecticide chlordecone (CLD). After 48 h, males and females (400 individuals each) were sorted for RNA extraction. In total, 2566 different genes were differentially expressed after EDC exposures compared to controls with similar numbers of DE genes with both compounds. More genes were differentially expressed in males than in females after both exposures [176].

Guo et al. evaluated the effects of effluents containing phenolic compounds from the Ba River on the ovary of the Sharpbelly (*Hemiculter leucisculus*), a freshwater fish using transcriptomic and metabolomic analyses. In fish collected near wastewater discharge, oocyte development was activated, compared to upstream and remote sites. Histopathological alterations were found in the fish ovaries likely a result of upregulated steroid hormone biosynthesis, as suggested by the differentially expressed genes from the RNAseq [177].

## 5. Risk Assessment Based on Genomic Technologies

A risk assessment, in the context of public health, refers to the process of identifying how detrimental to human health an activity or substance is, and then establishing the likelihood of any impact on human health. For example, acrylamide is regulated in drinking water by the US Environmental Protection Agency (EPA) in America. They consider the Maximum Containment Level Goal (MCLG) for acrylamide to be 0 mg/L [178]. Risk assessments are an inherent component in drug development. Research by the Tufts Centre for the Study of Drug Development and published in the Journal of Health Economics estimated that in 2014, a new prescription medicine that gains marketing approval is estimated to cost drugmakers $2.6 billion [179]. This elevated cost, the long development period (typically a minimum of a decade), and the push for safer, cheaper drugs have provided an opportunity for toxicogenomics to play an essential role in reforming the preclinical toxicology screening of drugs [180,181].

The Open Toxicogenomics Project—Genomics Assisted Toxicity Evaluation Systems (TG-GATEs) is a database that has collected toxicology data, consisting of biochemical, hematological, and histopathological findings, combined with the pathology imaging from in vivo studies, and the cytotoxicity data of in vitro studies. These data profiles have been compiled for 170 compounds, at varying dosages and time points [182]. The data of the 170 compounds were collected by the Toxicogenomics Project (TGP), a project between both the Japanese government and the private sector, organized by the National Institute of Biomedical Innovation (NIBIO), the National Institute of Health Science (NIHS), and 18 pharmaceutical companies.

Data was generated for the TGP at NIHS, NIBIO, and several Contract Research Organizations (CRO) using Standard Operating Procedures (SOP), thus ensuring data collection was uniformly done so by different sources. The data was collected over a period of 10 years, and the results were stored, managed, and analyzed in a closed database, an offline version of TG-GATES. According to the EU directive 2003/63/EC, relating to medicinal products for human use, before a clinical trial can be conducted, there must take place pharmacological and toxicological tests, conducted on animals [183]. This legislative requirement ensures that animal testing is a constant factor in developing new medicines for human use. The team behind the Open TG-GATES database [182] developed the database with the intention of moving away from animal testing, arguing that the measurement of cytotoxicity as opposed to general toxicity, will allow for the detection of potential toxicities, which may not be observable using general toxicity assessments in rats.

The current process of animal testing is highly time consuming and comparatively expensive. The Human Society International estimates that a rat developmental toxicity test costs approximately $50,000 to conduct in animals, compared to a rat limb bud test, an equivalent in vitro test, which costs approximately $15,000, roughly 1/3 of the cost [184]. While there is some work being conducted on moving away from animal testing, informatics is a comparatively unexplored area, which is unfortunate, given the potential for huge cost savings. The most significant measure to consider is that experimental data only needs to be collected once if it adheres to FAIR data standards of findability, accessibility, interoperability, and reusability [185]. The TG-GATES database is comprised of gene expression profiles, and toxicological data from in vivo (rats), and in vitro (rat hepatocytes and primary human hepatocytes) sources. These sources were exposed to 170 compounds. For the in vitro studies, compounds were tested at up to four dose levels and three time points via duplicate microarray hybridization analysis [182]. For the in vivo, compounds were tested at up to four dose levels and eight time points, which corresponds to four single-dose studies, and four repeated dose studies [182]. Now that this data has been collected, the cost does not need to be repeated for further studies.

The Open TG-GATES database has a substantial library of EDCs, which include chemicals, such as detergents, and pesticides. Many EDCs and pharmaceutical or personal care products (PPCPs) enter water sources, where they can interact with fish, humans, and other animals, either through their consumption of contaminated drinking water, or the consumption of other animals that have consumed the contaminated water.

The Water Quality Association lists several potential entry points for EDCs into the water supply, including elimination from the body, disposal of expired or unused drugs via landfill, and drainage during showering/bathing are common pathways [186]. For agricultural/veterinary EDCs, runoff to surface water/groundwater reserves is another common pathway [186]. Estimates indicate that up to 90% of oral drugs pass through the human body and enter the water supply [186].

In assessing the presence of EDCs it is important to consider that as physiologically active chemicals, they are intended to have an effect. The question, instead, is to consider the concentrations these chemicals in the water source, and whether it is possible for the EDCs to induce the intended effect, or any effect, at these concentrations [186]. The current process used for risk evaluation is to calculate the acceptable daily intake (ADI), or tolerable daily intake (TDI). This involves the use of the minimum therapeutic dose (MTD) or no-observed-adverse-effect-level/lowest-observed-adverse-effect-level (NOAEL/LOAEL), alongside an uncertainty factor, which is used to account for uncertainty in the accuracy of test results, and difficulties in estimating health effects in different species/exposure conditions [187]. The uncertainty factor acts as a safety buffer, making the final number conservative, erring on the side of caution to account for uncertainty in the test result accuracy. From this, a drinking water effect level (DWEL) can be calculated, which is compared to occurrence data to establish safety margins [186]. One of the reasons EDCs are such a concern for environmental groups and government agencies is their chemical similarity to other physiological compounds. As noted above, BPA has structural and chemical properties like estradiol, allowing it to bind to an ER and disrupt estrogen physiology in humans, crustaceans and fish [188].

The increase in available datasets and computational capacity has also made it possible to efficiently use artificial intelligence approaches in genomics, giving biomedical research a new predictive role [189,190]. The biggest challenge to implementing machine and deep learning approaches in toxicogenomics mainly lies in the nature of the available data, characterized by high dimensionality and a low number of samples. The increasing number of available datasets, particularly from public databases, such as DrugMatrix [191], Gene Expression Omnibus (GEO) [192], The Expression Atlas [193] and the Comparative Toxicogenomics Database (CTD) [194] will address the problem of low sample counts. Furthermore, the development of panels designed specifically for toxicogenomics studies, such as LINCS100 [195] and S1500+ [196] could help reduce the dimensionality of these datasets, finally unlocking the efficient use of artificial intelligence in toxicogenomics.

## 6. The Adverse Outcomes Framework

**Figure 1:**
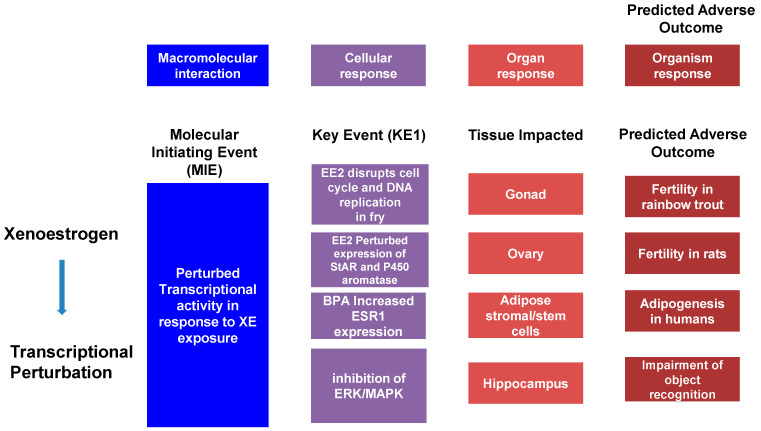
The adverse outcome pathway (AOP) is a linear pathway composed of a Molecular Initiating Event (MIE), Key Events (KE), and an Adverse Outcome (AO) causally linked together. Example AOPs are illustrated.

The Adverse Outcomes Framework (AOF), developed in 2007 with the release of a National Research Council report entitled “Toxicity Testing in the 21st Century,” has become a popular model in the study of toxicology. This model defines an Adverse Outcome Pathway (AOP) as a linear pathway comprised of a molecular initiation event (MIE), key events (KE), and the Adverse Outcome (AO) itself causally linked together (Figure 1). Using cancer as an example, the MIE would be a single mutation in a gene associated with, for example, control of the cell cycle. This, alone, does not cause cancer but may trigger pathway level perturbations, so-called key events, that could reduce the expression of tumor-suppression genes and mutate proto-oncogenes into oncogenes. The goal of this framework is to transform current toxicology testing by supporting less animal-intensive alternatives to toxicity testing and predictive ecotoxicology [197,198].

Adverse Outcomes are not always the result of a single event. Cancer, for instance, is more common among adults as they age due to genomic damage including point mutations and insertion-deletions that disrupt normal cell function, allowing for the uncontrolled growth and division of cells. The first of these mutations is defined as the MIE, which is then followed by other mutations—KE—that are linked by KE relationships (KERs) and further alter expression in such a way that an adverse outcome is the result. Going beyond the simple, linear AOPs are AOP Networks, quantitative AOPs (qAOPs), and qAOP Networks. An AOP network is a system of interacting AOPs with shared KEs, while qAOPs are AOPs in which the KERs (qKERs) are given a weight/value that quantitatively characterizes the relationship between KEs in an AOP. The AOF identifies qAOPs as the “ideal” form to be used in risk assessment due to this quantification [197,198].

To help make the AOF easier to comprehend, Villeneuve et al., developed a set of principles that summarize the development of this model [199]: (1) because any external stress or chemical that can trigger an MIE has the potential to activate a chain of KEs leading to the AOP; AOPs are not specific to given chemicals. (2) AOPs are composed of two fundamental units: KEs and KERs that are usually shared between AOPs. (3) AOPs are not a thorough interpretation of biological processes, but a basic and organized framework for organizing toxicological evidence; this defines individual AOPs as rational units of AOP development. (4) Those networks compiled of numerous AOPs are likely the most functional units of prediction in everyday scenarios, as the prediction of adverse outcomes will often involve contemplation of multiple AOPs. Finally, (5) AOPs are not stationary and will evolve as new insights are offered [197,199].

As the AOP is a developing concept there are some limitations to the framework that must be addressed to improve its viability. One of the most critical considerations is the ability of AOPs to accurately define scientifically robust connections between the MIE, KEs, and adverse outcomes [197]. The application of AOP networks and qAOPs, described above, will facilitate more accurate predictions, but at present, there remain gaps between these events. It has been suggested that, for AOPs to be useful as more than a categorizing tool, they must increase trust for risk decisions more than existing approaches, and must be able to reduce animal usage as well [198]. In short, the AOP framework will need to demonstrate, beyond a shadow of a doubt, that it is effective at predicting adverse outcomes in order for it to be accepted by the scientific community [197].

As outlined above, EDCs have become common in the environment; though there are efforts to reduce these compounds humans are still potentially exposed to these chemicals. The studies above utilize two approaches for determining the toxicity of these EDCs: using toxicological concentrations of XEs or using levels found in the environment, in other words, the concentrations most people will be exposed to daily. The AOP framework establishes that the development of an adverse outcome is not necessarily completed overnight; rather, a linear series of key events following the initial MIE create a stockpile of genetic damage that, later in life, impact expression in such a way that the adverse outcome is the result.

## 7. Conclusions

To conclude, endocrine disrupting compounds are ubiquitous in modern life. BPA, for example, is an industrial chemical used in the fabrication of plastics and found in the urine samples of up to 96% of Americans. It is estimated that 1000 of the 85,000 synthetic chemicals in existence may be EDCs, however, the majority of these have not undergone sufficient testing to confirm this, so the true number may be much higher [200]. In addition there are a large number of chemicals that have yet to be fully profiled to determine their capacity to act as EDCs. The cost of profiling each chemical is not insignificant. Genomic and bioinformatics approaches have revolutionized toxicology testing, permitting deep insights into the transcriptomes of cells and tissues following exposure to EDCs and reducing the costs that are associated with traditional toxicity testing. Genomic profiling linked to computational approaches, such as The Open TG-GATES database presents an opportunity to avoid the cost associated with profiling each chemical for its efficacy as an EDC and to accelerate the process using high-throughput informatics. The Adverse Outcome Pathway (AOP) framework integrates these genomic and computational outputs and allows a molecular initiation event, key events resulting from this initial insult, and the Adverse Outcome (AO) itself to be causally linked together in the context of predictive toxicology assessments.

## Data Availability

Not applicable.
